# Supplementary figures and images for: Influence of Melt-Pool Stability in 3D Printing of NdFeB Magnets on Density and Magnetic Properties
Source: Materials (Basel). 2019 Dec 29;13(1):139. doi: 10.3390/ma13010139 (PMC6981518; doi:10.3390/ma13010139)

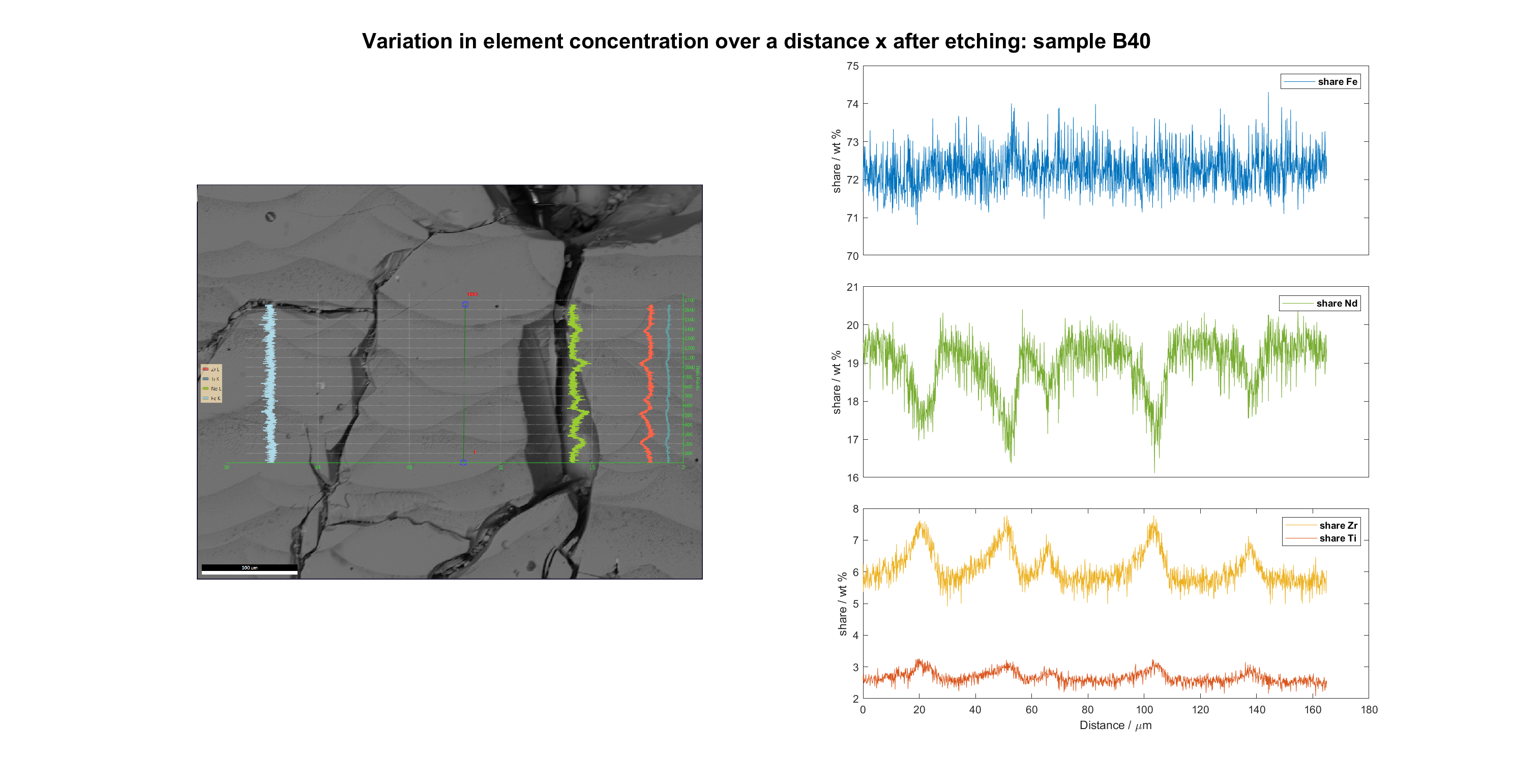

Supplement: Supplementary file 1 [file materials-13-00139-s001.zip › supplementary data/EDAX linescans/B40/B40_with etching.png]

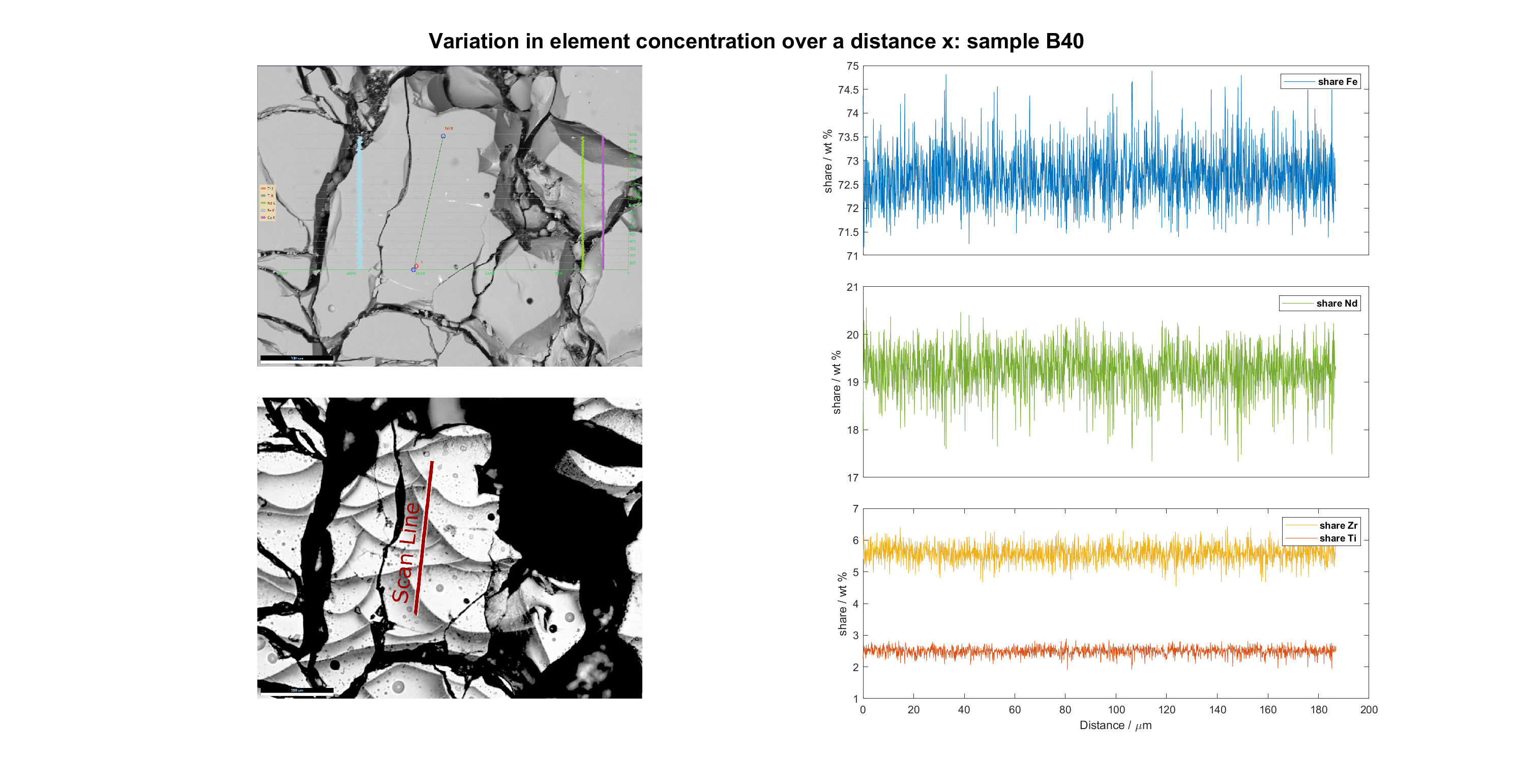

Supplement: Supplementary file 1 [file materials-13-00139-s001.zip › supplementary data/EDAX linescans/B40/B40_without etching.png]

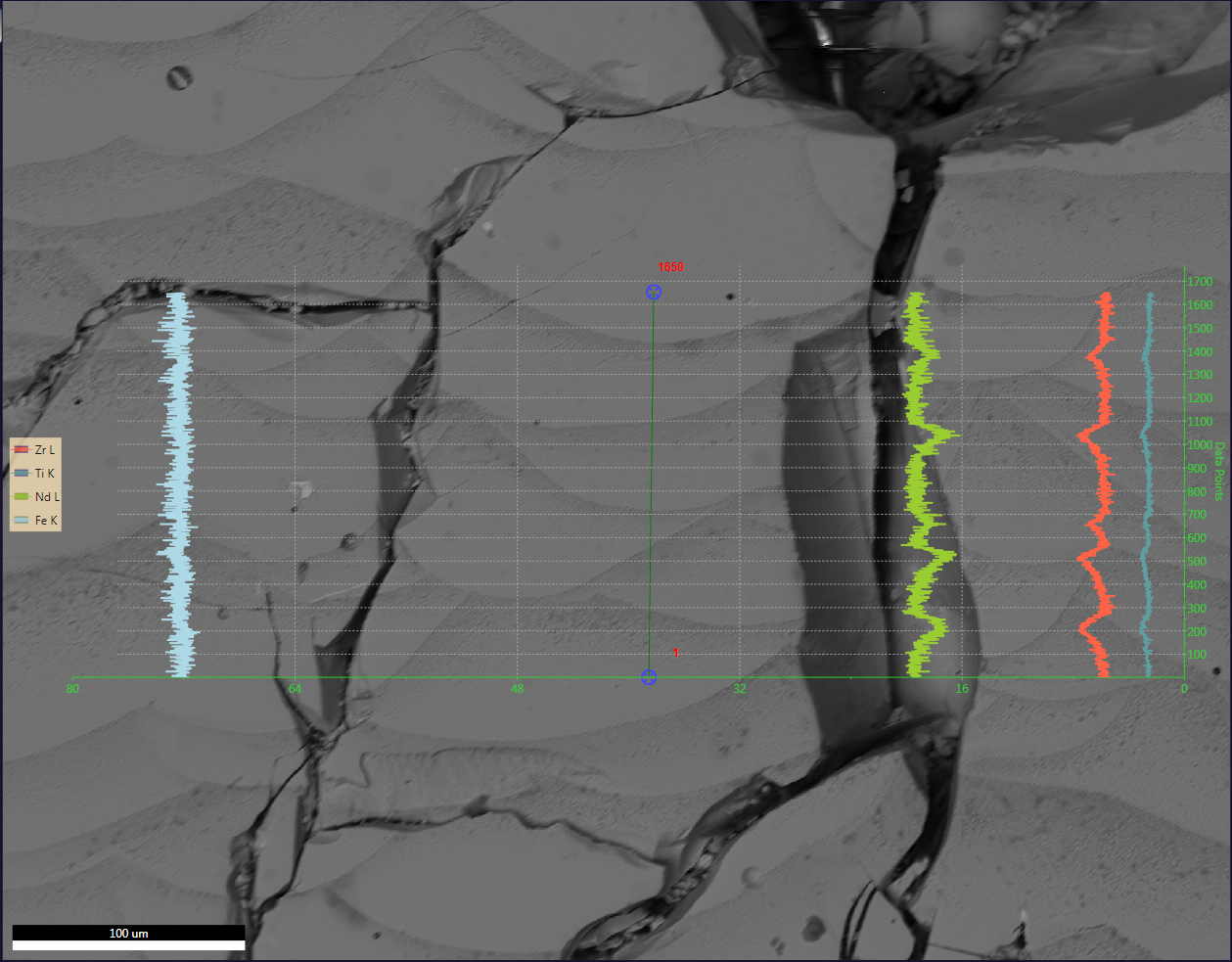

Supplement: Supplementary file 1 [file materials-13-00139-s001.zip › supplementary data/EDAX linescans/B40/Map_mitÄtzen.PNG]

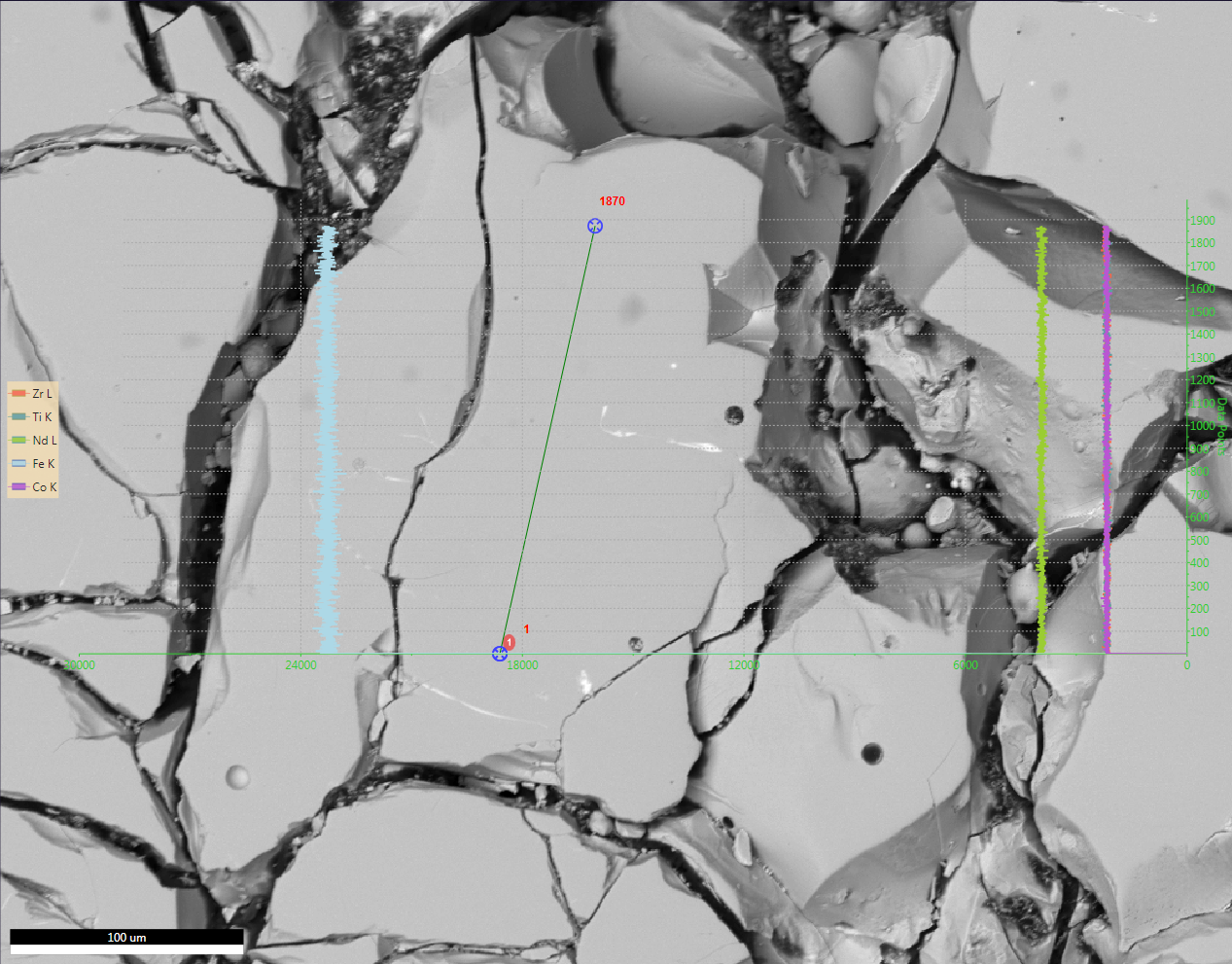

Supplement: Supplementary file 1 [file materials-13-00139-s001.zip › supplementary data/EDAX linescans/B40/Map_ohneÄtzen.PNG]

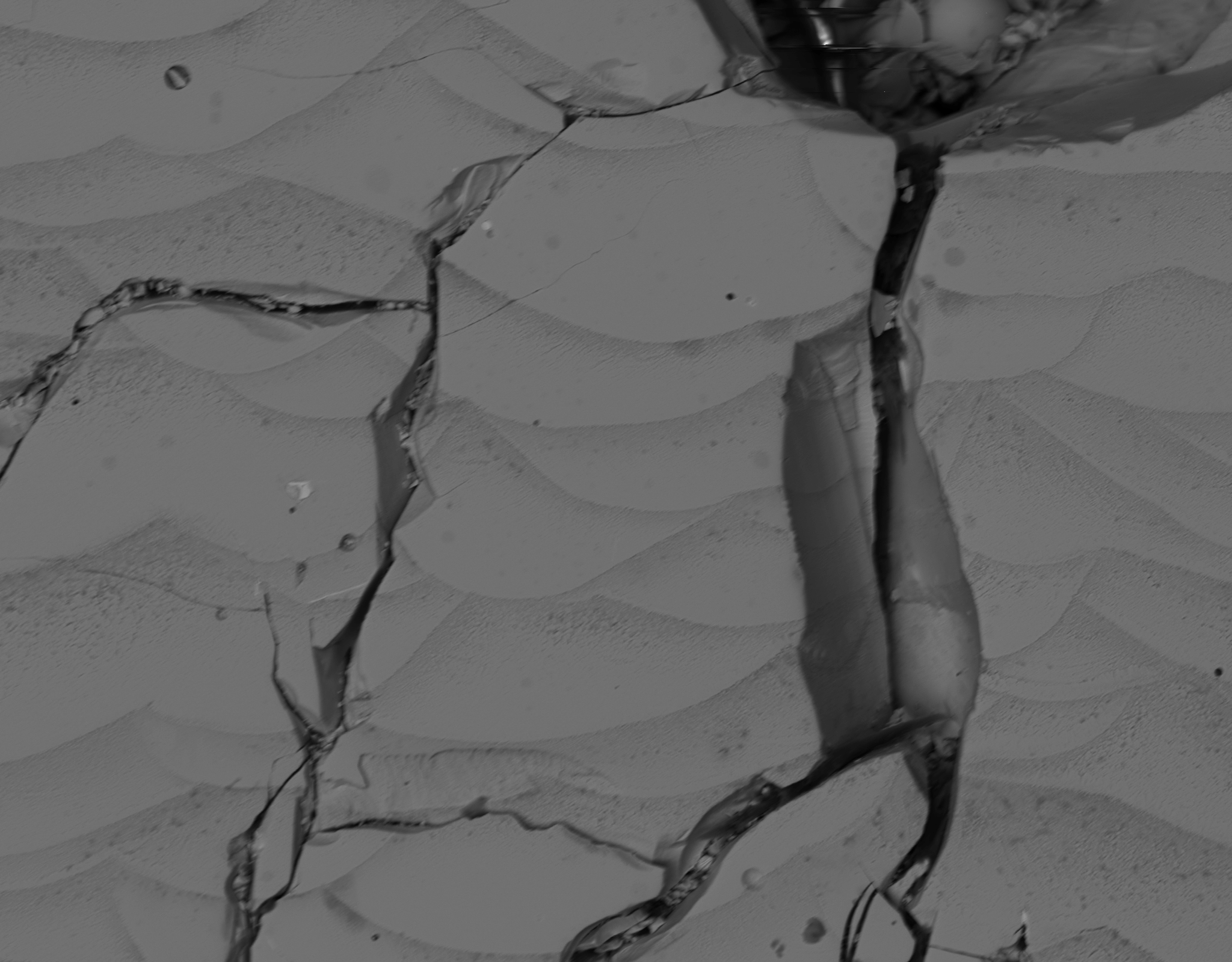

Supplement: Supplementary file 1 [file materials-13-00139-s001.zip › supplementary data/EDAX linescans/B40_with etching.bmp]

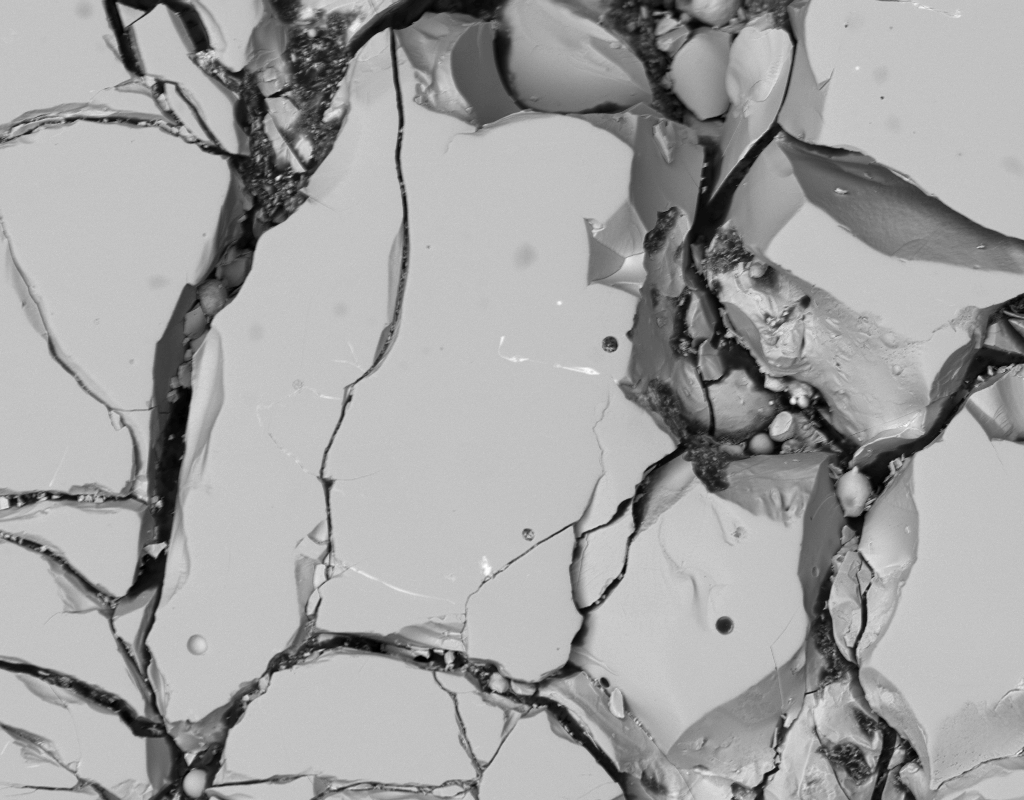

Supplement: Supplementary file 1 [file materials-13-00139-s001.zip › supplementary data/EDAX linescans/B40_without etching.bmp]

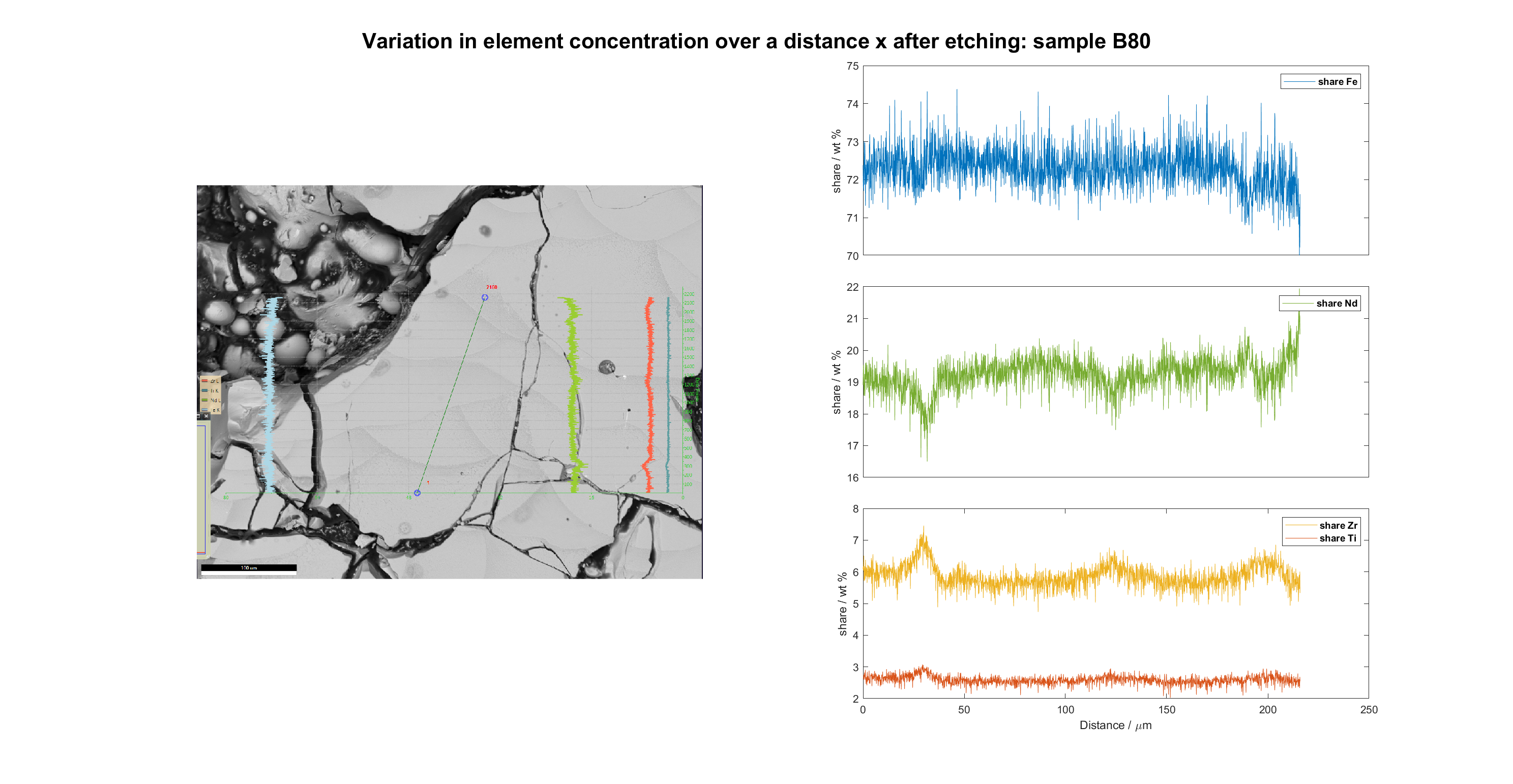

Supplement: Supplementary file 1 [file materials-13-00139-s001.zip › supplementary data/EDAX linescans/B80/B80_with etching.png]

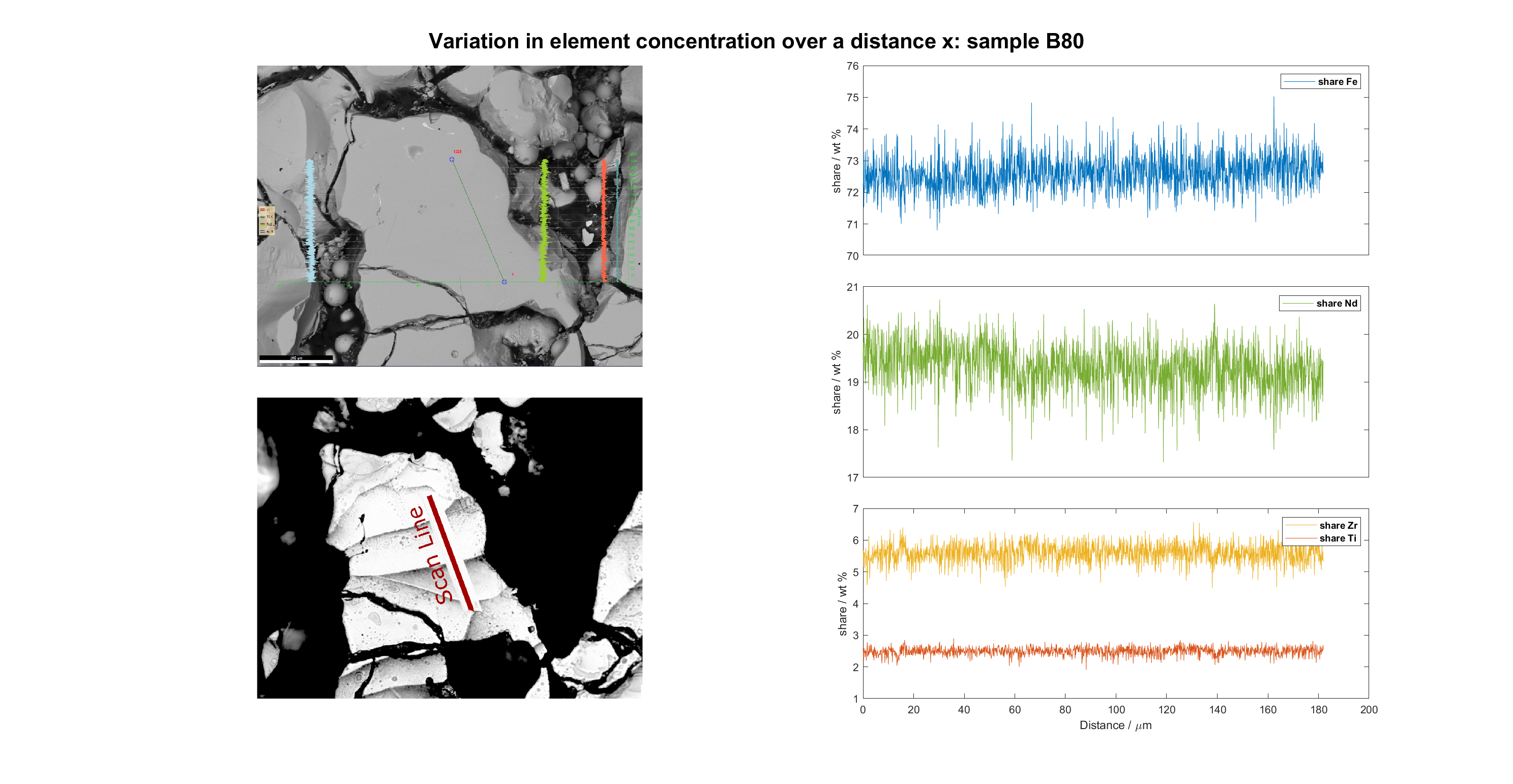

Supplement: Supplementary file 1 [file materials-13-00139-s001.zip › supplementary data/EDAX linescans/B80/B80_without etching.png]

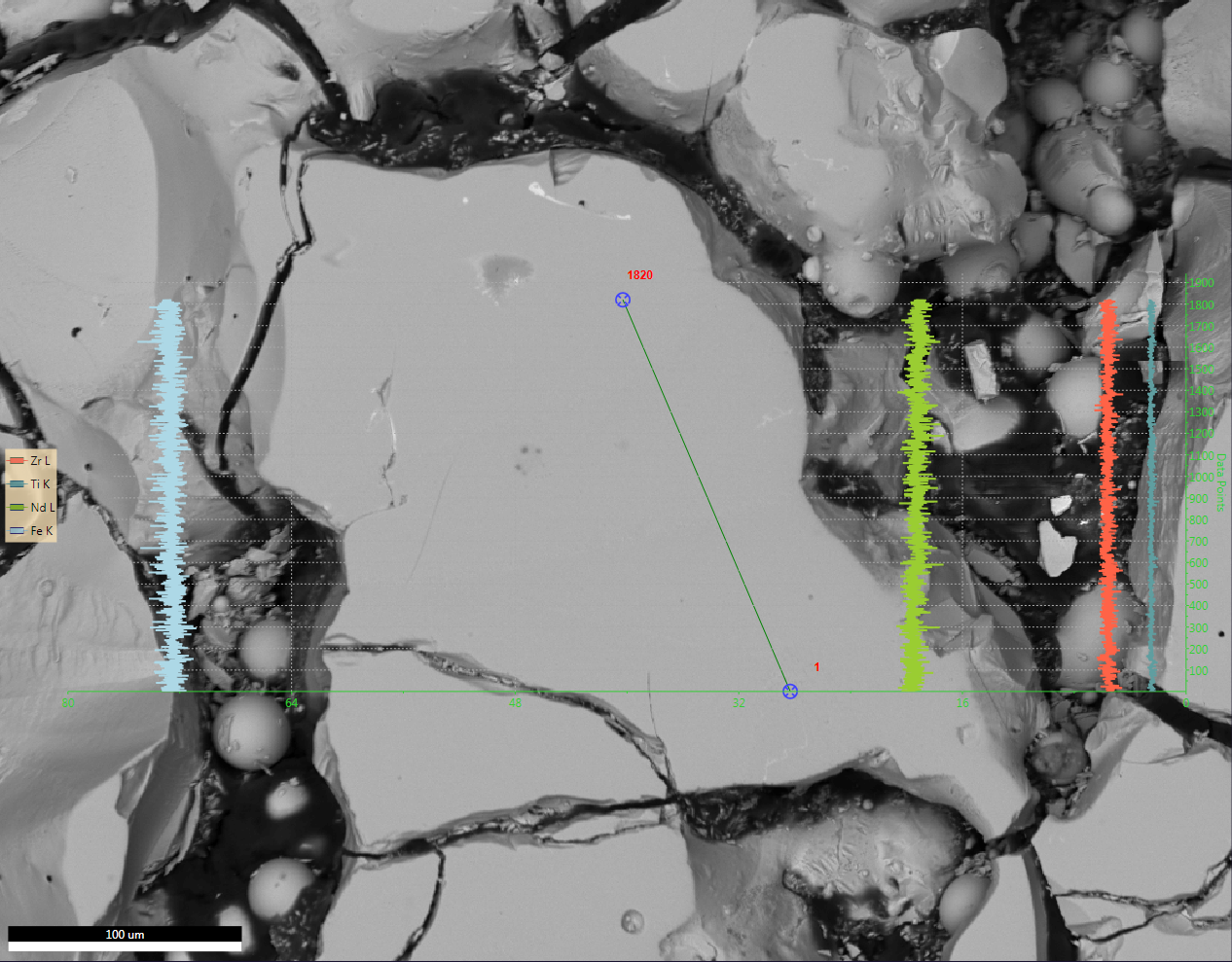

Supplement: Supplementary file 1 [file materials-13-00139-s001.zip › supplementary data/EDAX linescans/B80/Map2_ohneÄtzen.png]

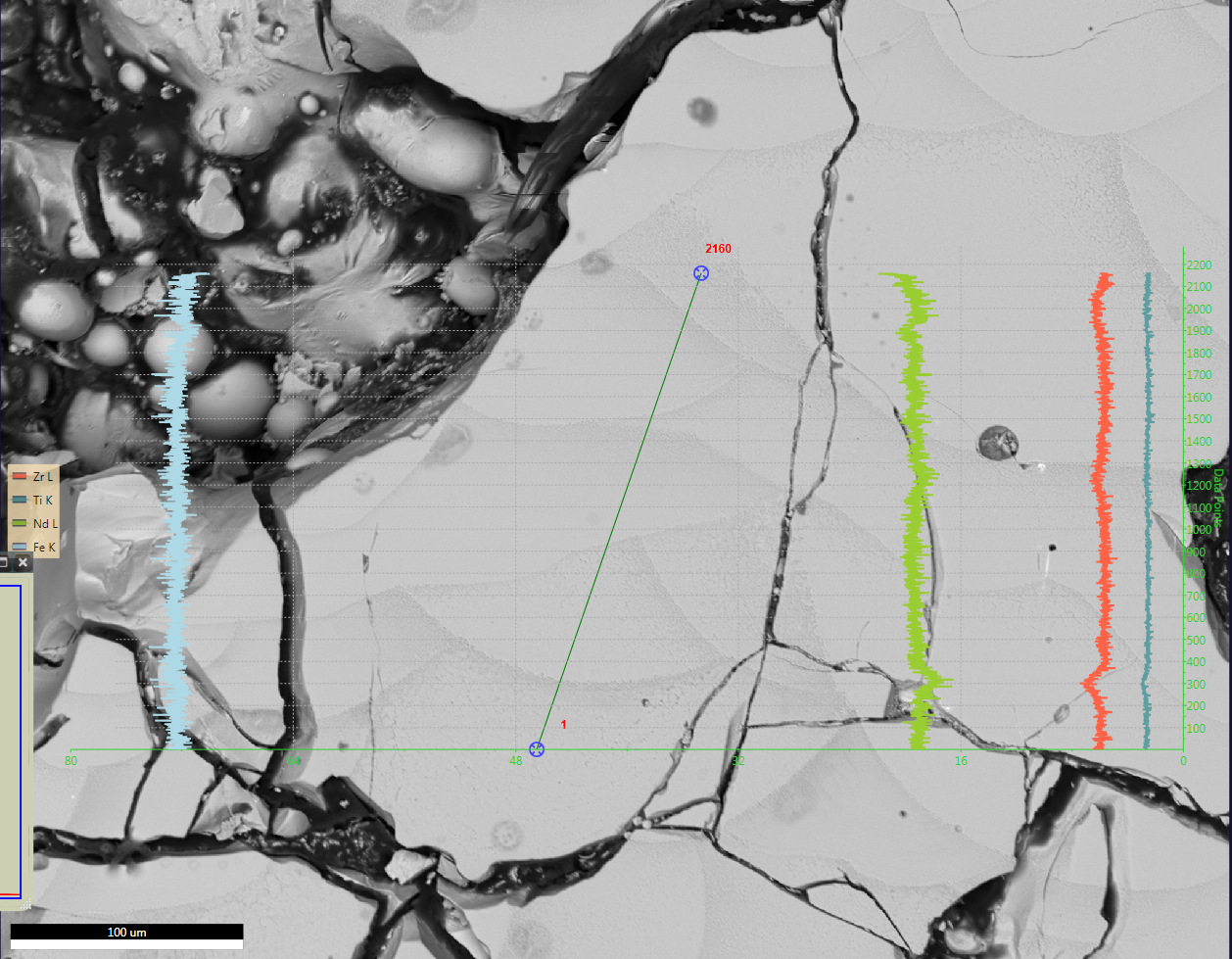

Supplement: Supplementary file 1 [file materials-13-00139-s001.zip › supplementary data/EDAX linescans/B80/Map_mitÄtzen.png]

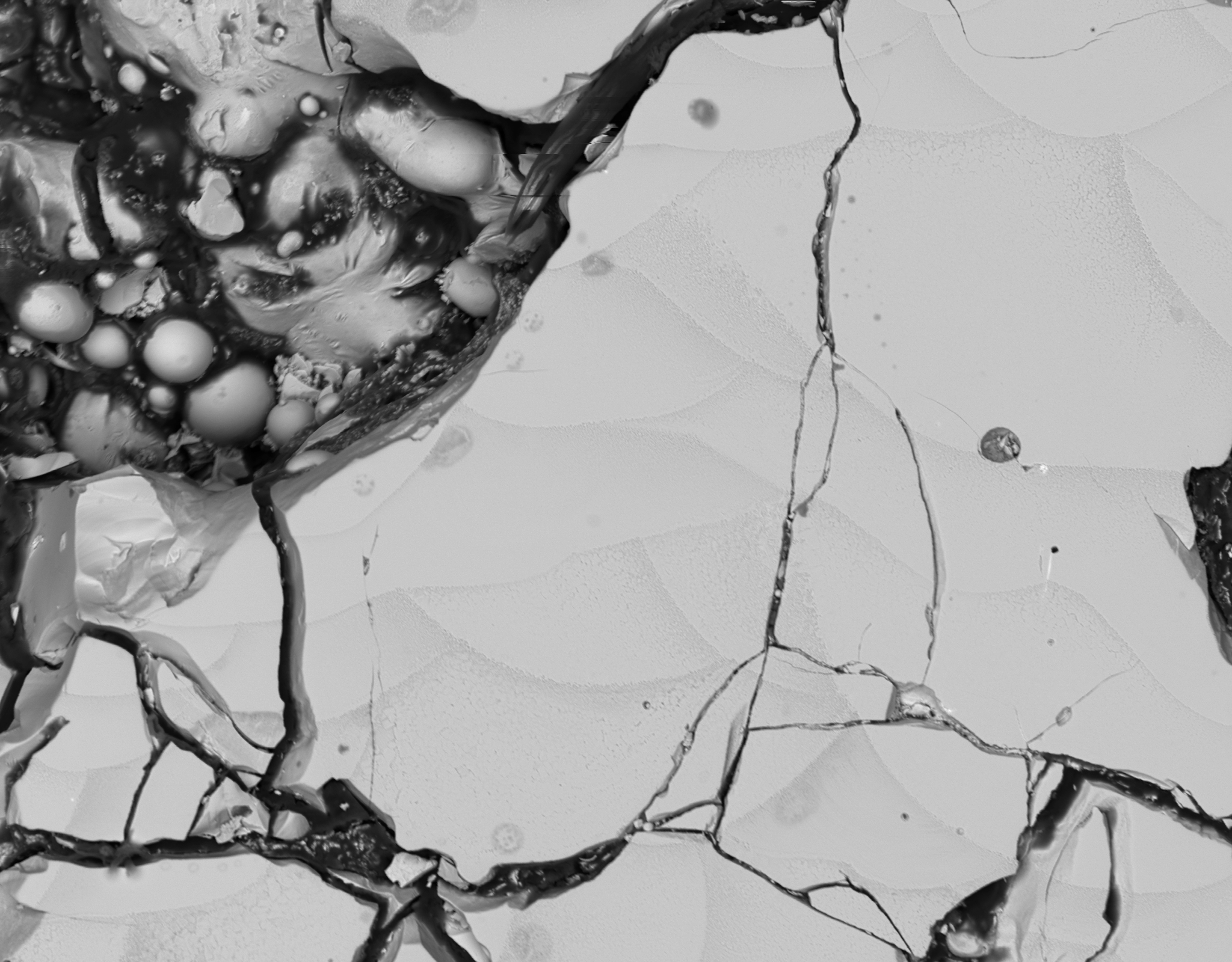

Supplement: Supplementary file 1 [file materials-13-00139-s001.zip › supplementary data/EDAX linescans/B80_with etching.bmp]

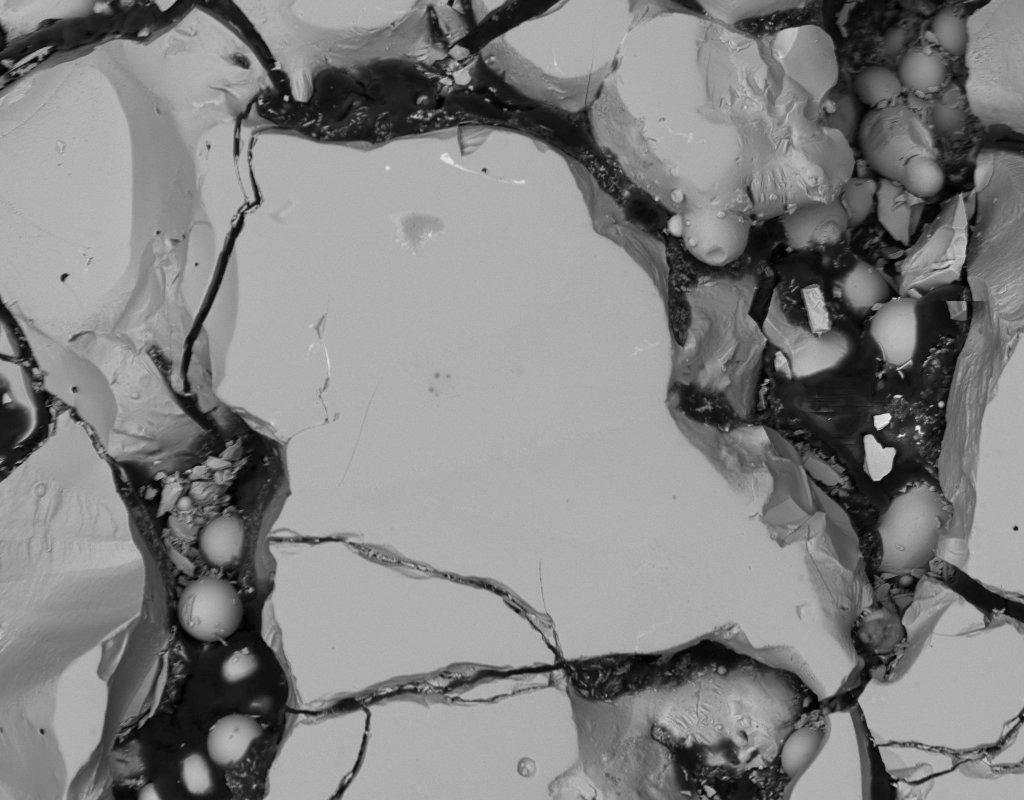

Supplement: Supplementary file 1 [file materials-13-00139-s001.zip › supplementary data/EDAX linescans/B80_without etching.bmp]

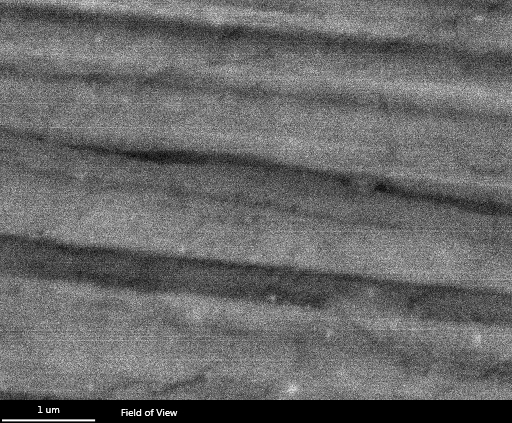

Supplement: Supplementary file 1 [file materials-13-00139-s001.zip › supplementary data/EDAX mapping/B60 EDAX mapping/Name_16.jpg]

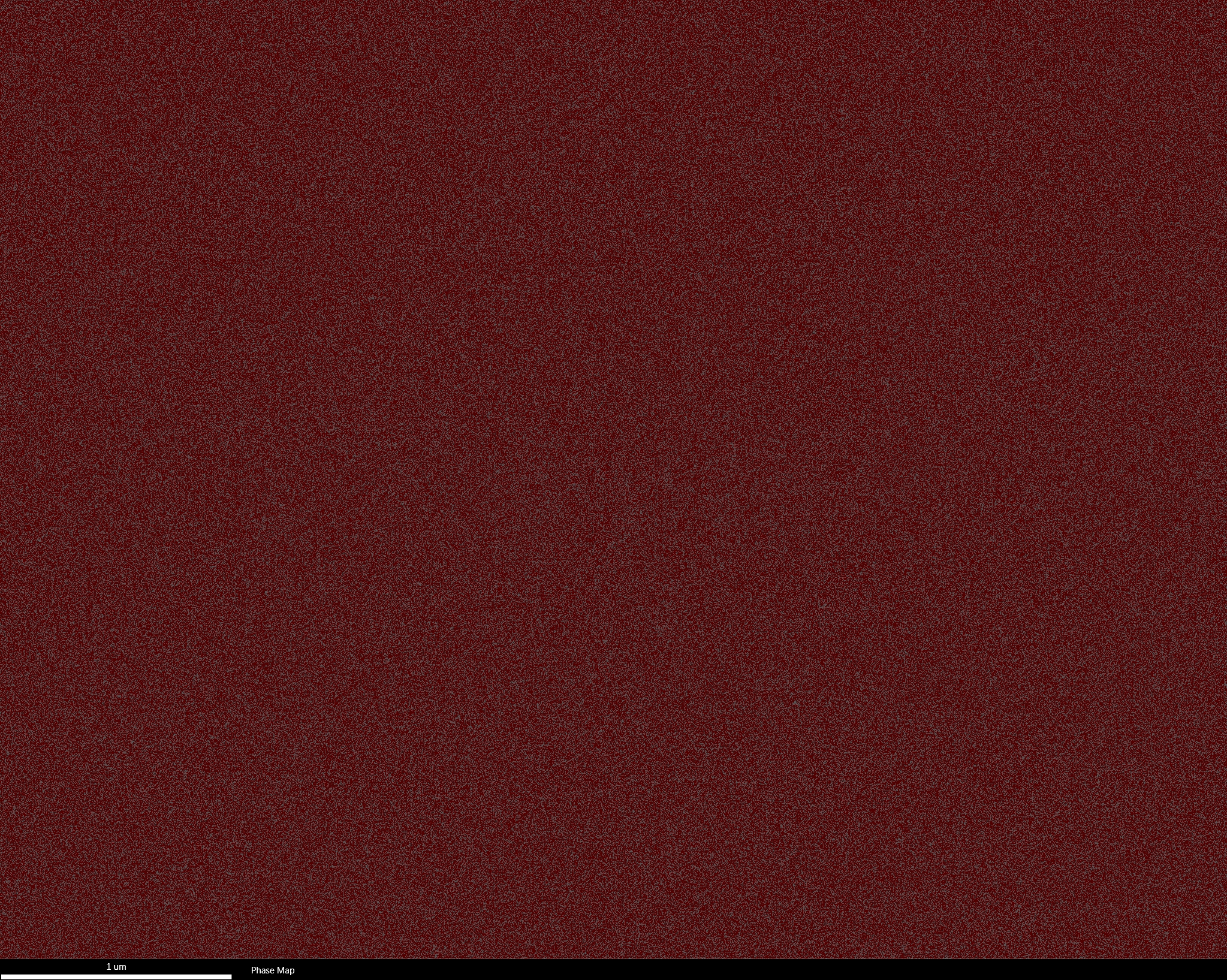

Supplement: Supplementary file 1 [file materials-13-00139-s001.zip › supplementary data/EDAX mapping/B60 EDAX mapping/Name_17.jpg]

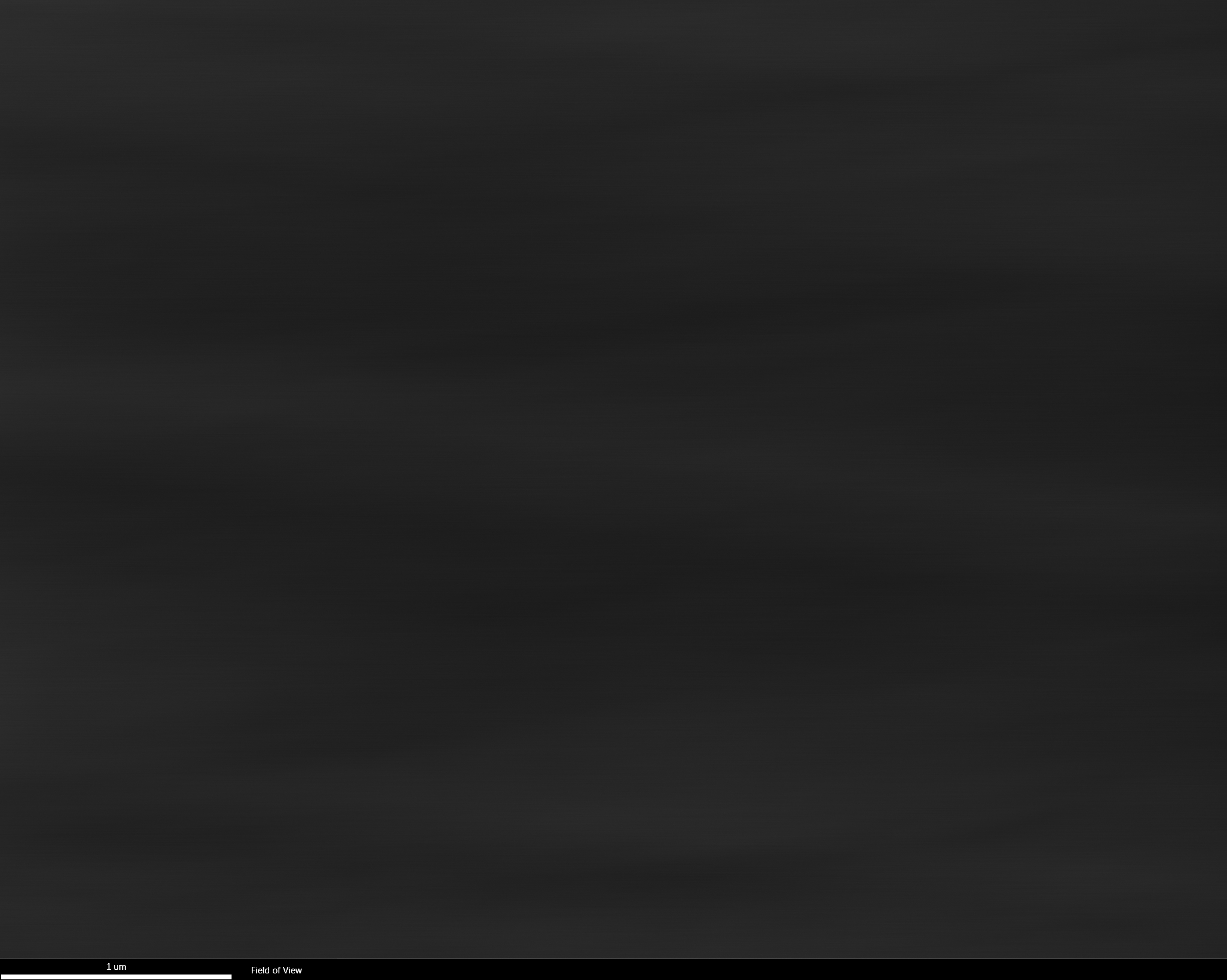

Supplement: Supplementary file 1 [file materials-13-00139-s001.zip › supplementary data/EDAX mapping/B60 EDAX mapping/Name_31.jpg]

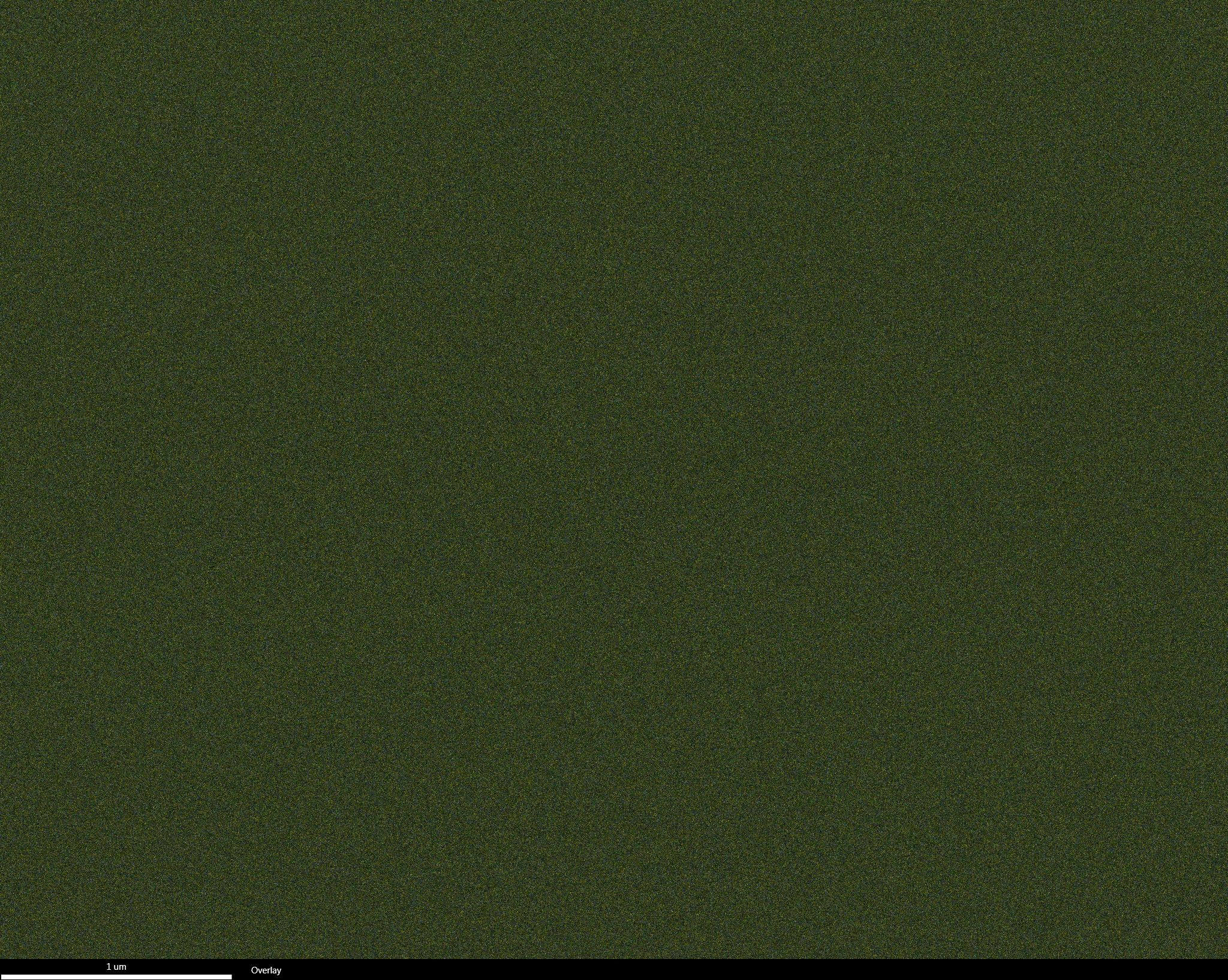

Supplement: Supplementary file 1 [file materials-13-00139-s001.zip › supplementary data/EDAX mapping/B60 EDAX mapping/Name_32.jpg]

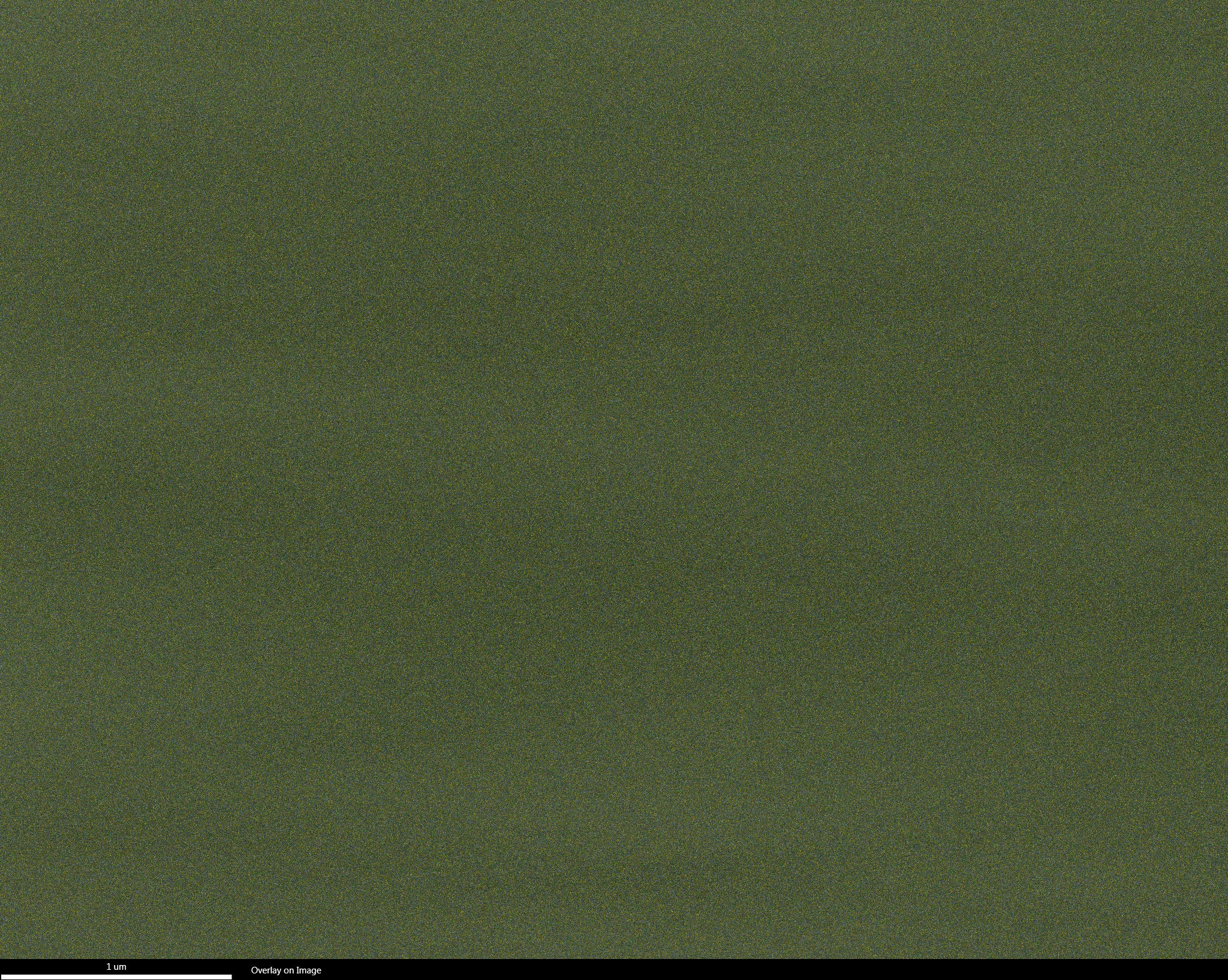

Supplement: Supplementary file 1 [file materials-13-00139-s001.zip › supplementary data/EDAX mapping/B60 EDAX mapping/Name_33.jpg]

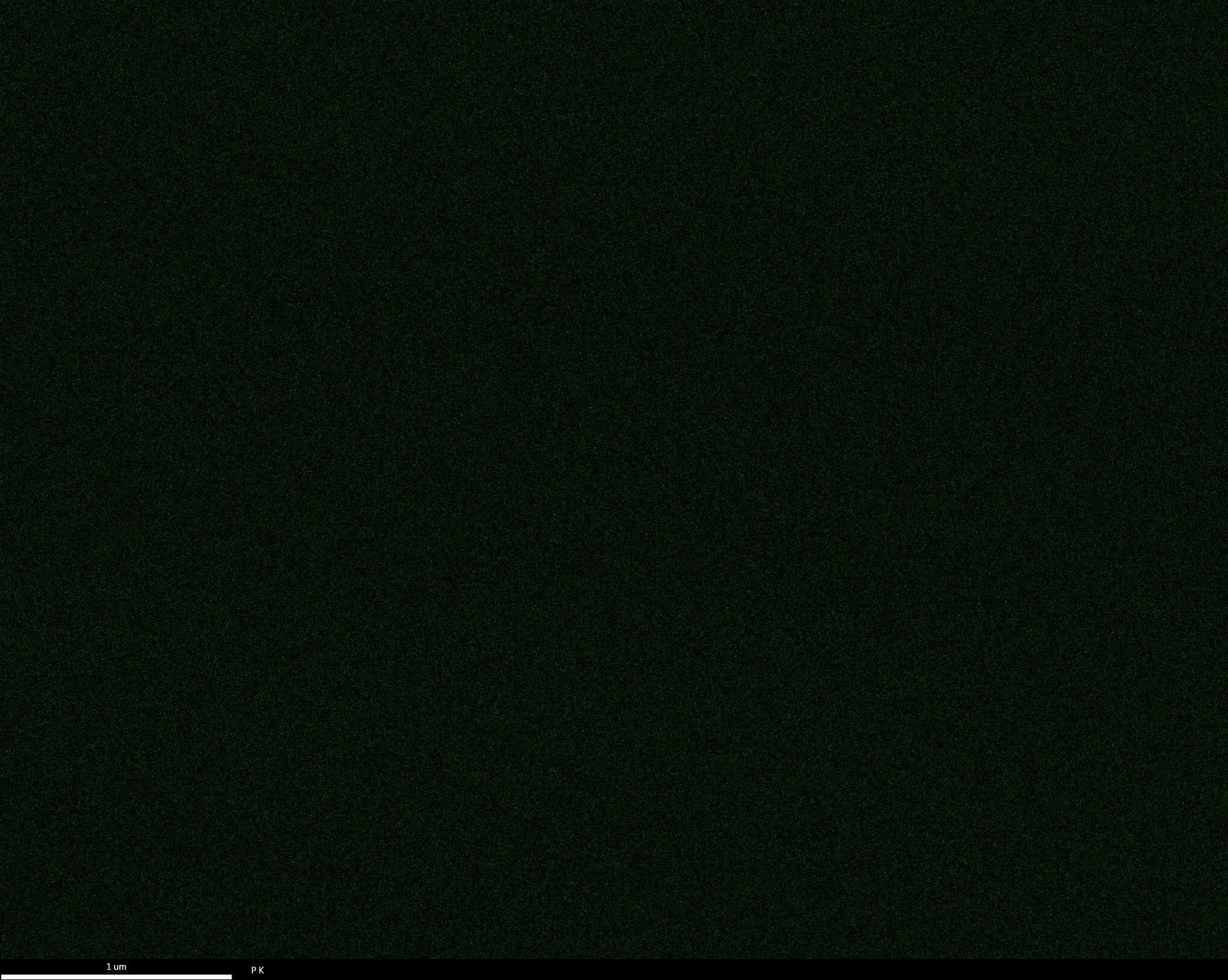

Supplement: Supplementary file 1 [file materials-13-00139-s001.zip › supplementary data/EDAX mapping/B60 EDAX mapping/Name_34.jpg]

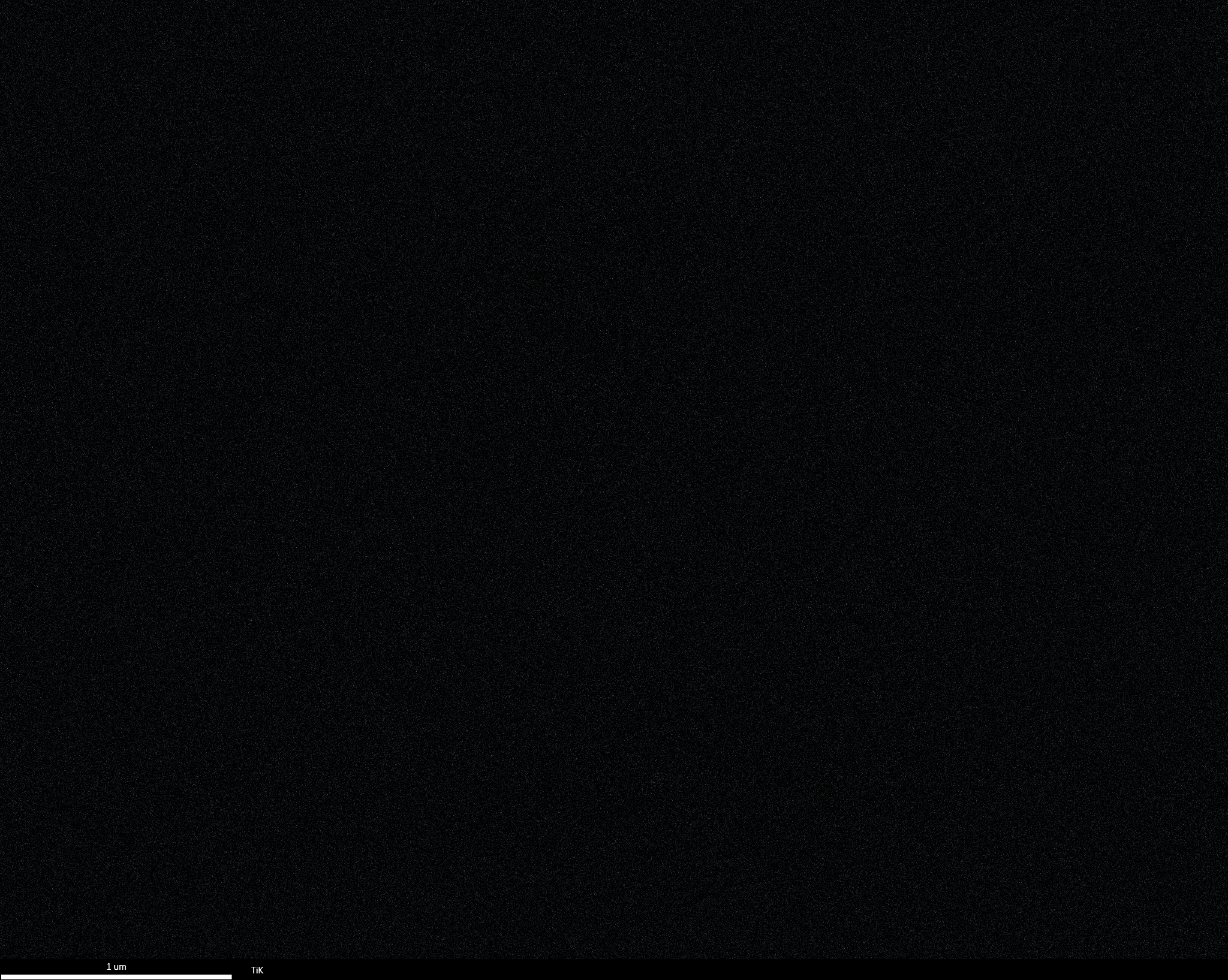

Supplement: Supplementary file 1 [file materials-13-00139-s001.zip › supplementary data/EDAX mapping/B60 EDAX mapping/Name_35.jpg]

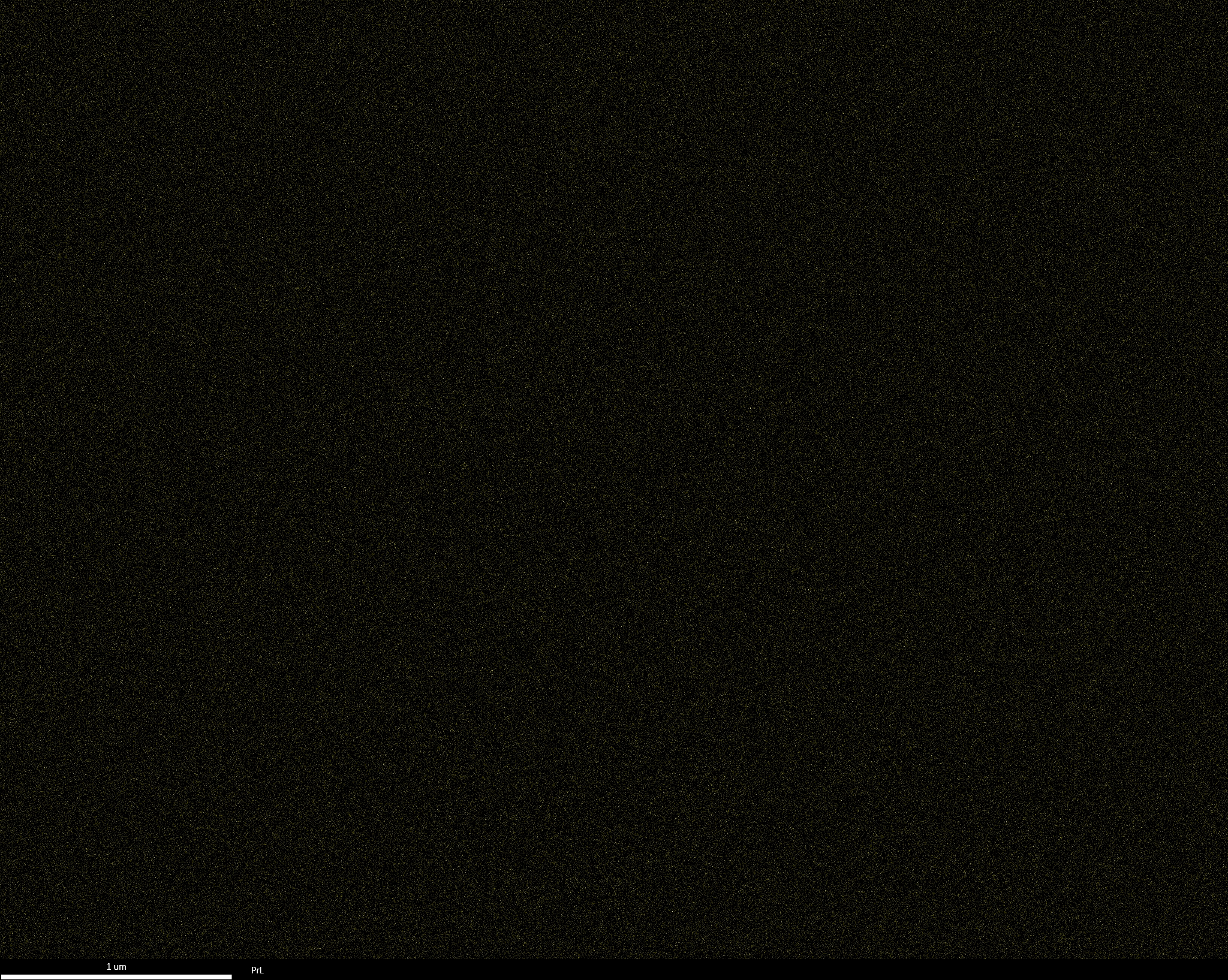

Supplement: Supplementary file 1 [file materials-13-00139-s001.zip › supplementary data/EDAX mapping/B60 EDAX mapping/Name_36.jpg]

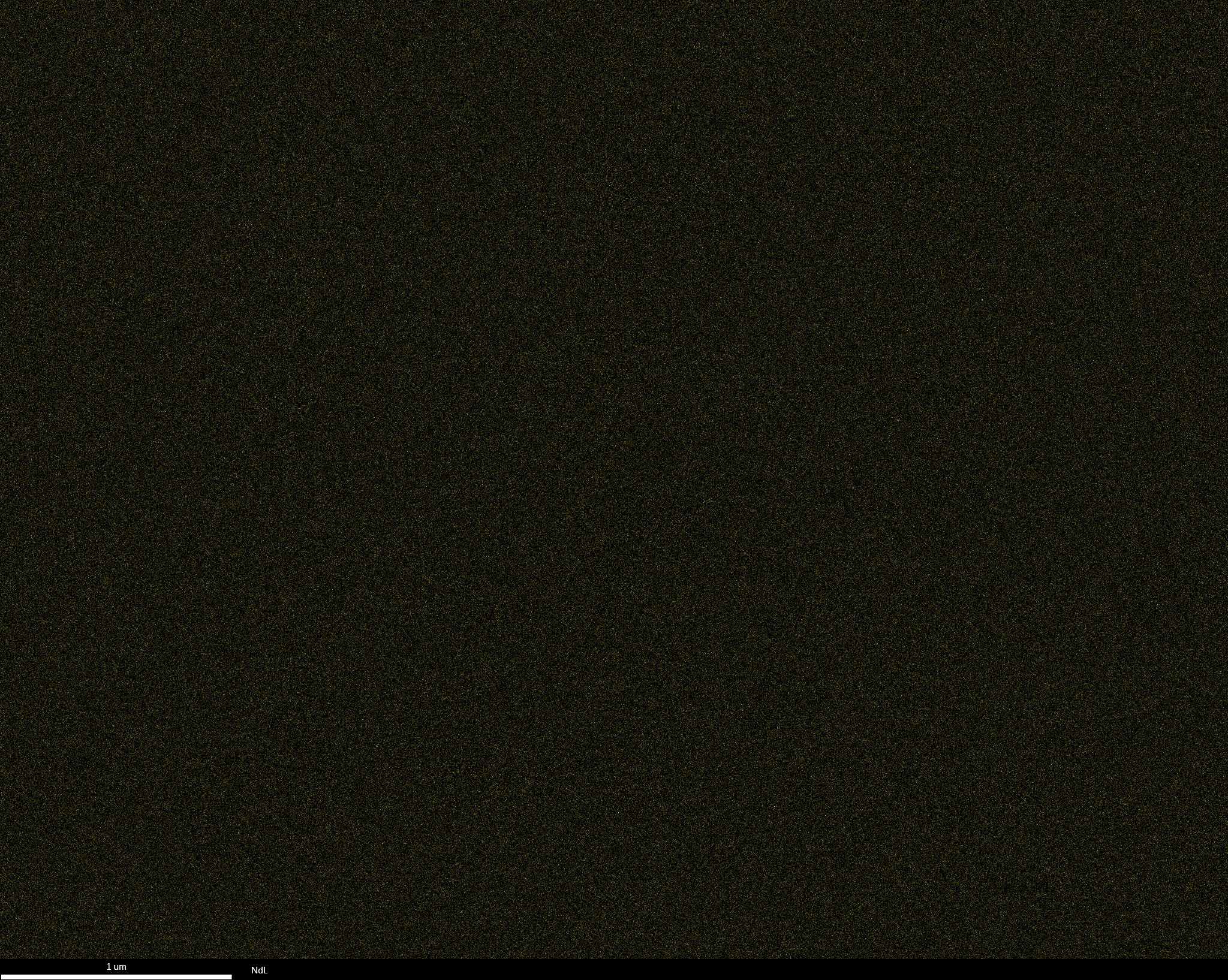

Supplement: Supplementary file 1 [file materials-13-00139-s001.zip › supplementary data/EDAX mapping/B60 EDAX mapping/Name_38.jpg]

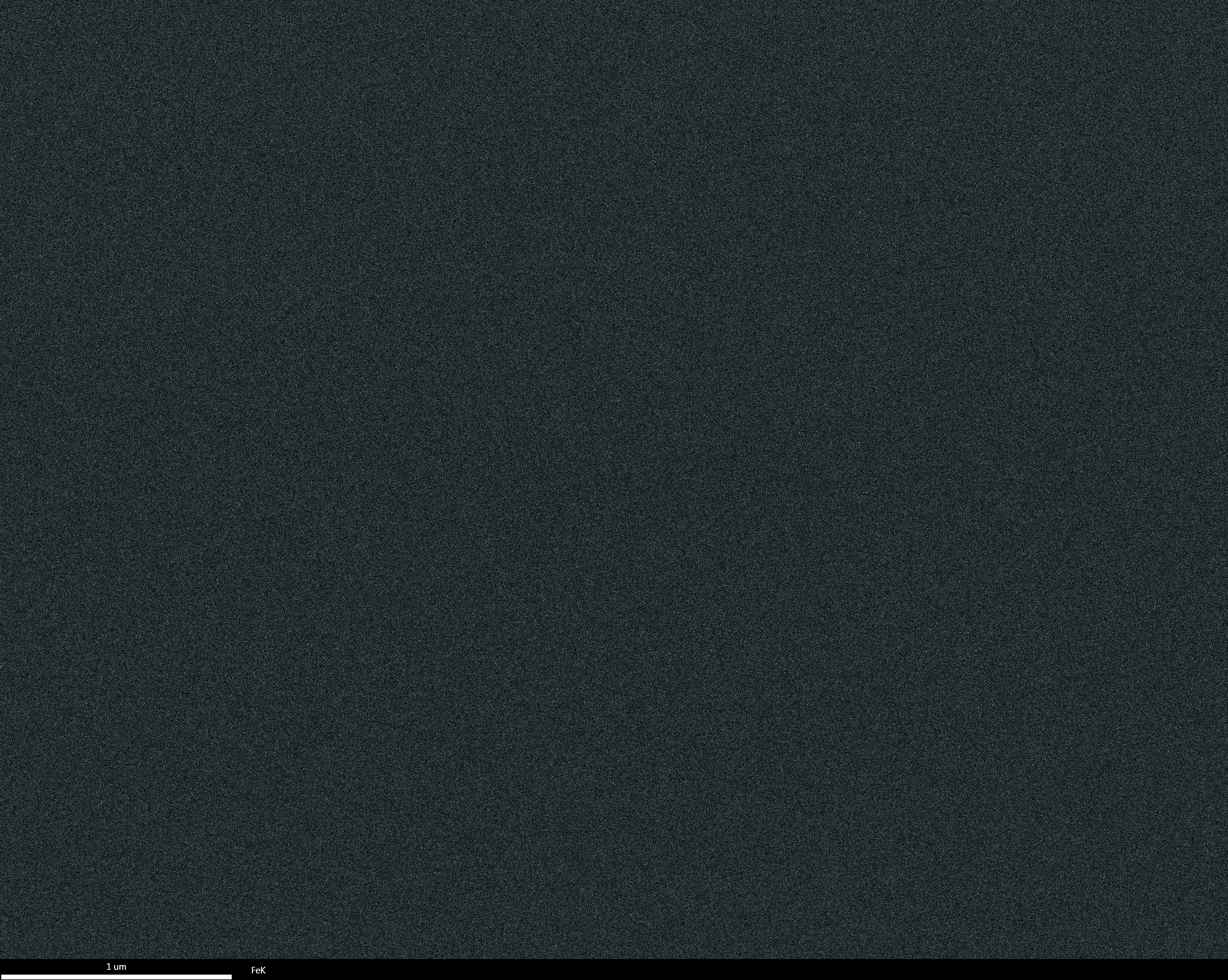

Supplement: Supplementary file 1 [file materials-13-00139-s001.zip › supplementary data/EDAX mapping/B60 EDAX mapping/Name_39.jpg]

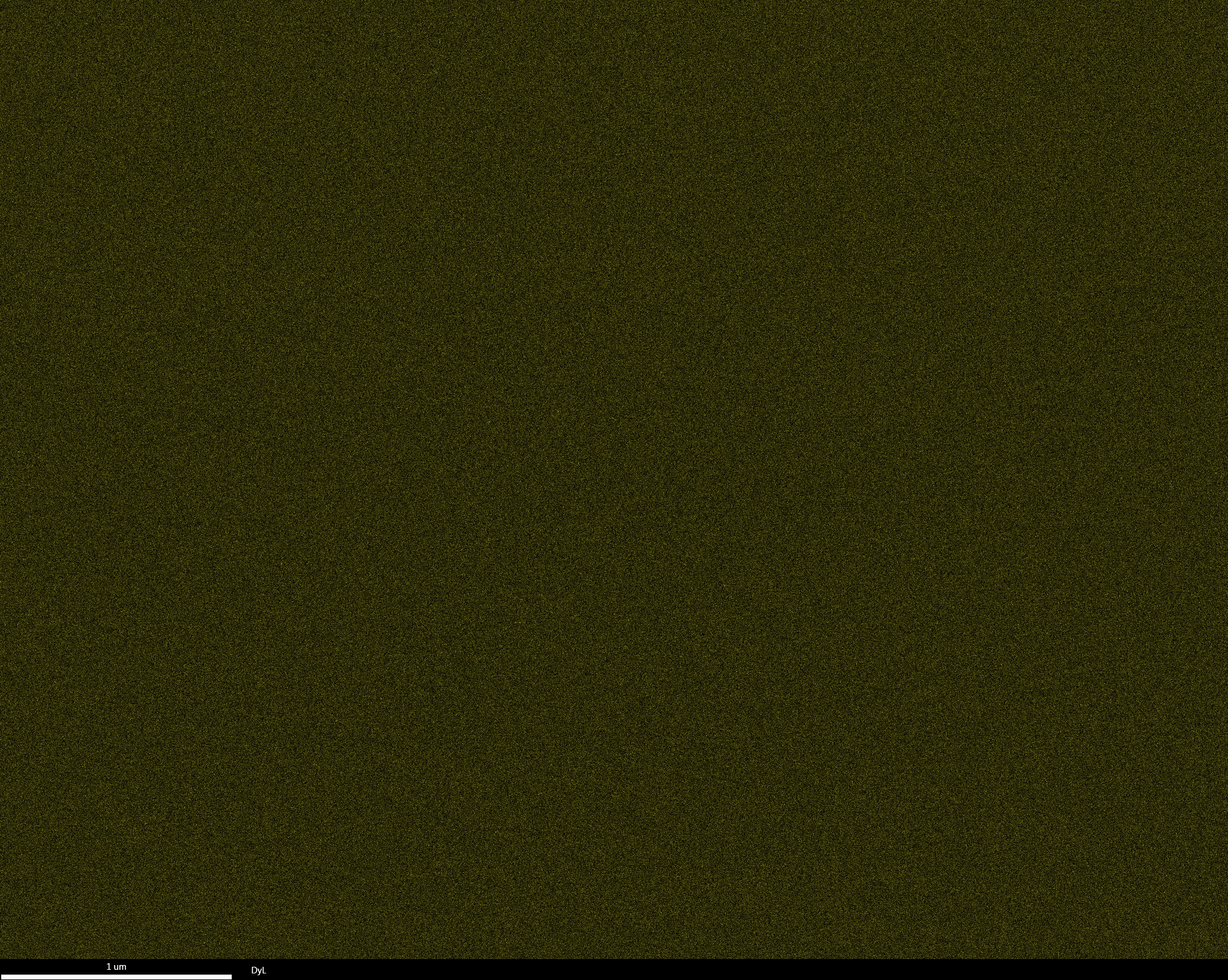

Supplement: Supplementary file 1 [file materials-13-00139-s001.zip › supplementary data/EDAX mapping/B60 EDAX mapping/Name_40.jpg]
